# Effects of Just Culture and Empowerment on Patient Safety Activities of Hospital Nurses

**DOI:** 10.3390/healthcare9101324

**Published:** 2021-10-04

**Authors:** Bo Bae Kim, Soyoung Yu

**Affiliations:** 1 Bundang CHA Women’s Hospital, Seongnam 13497, Korea; saturn@chamc.co.kr; 2 College of Nursing, CHA University, Pocheon 11160, Korea

**Keywords:** patient safety, hospital, nurses, culture, empowerment

## Abstract

This study investigated the relationship among hospital nurses’ perceptions of a just culture, empowerment, and patient safety activities. It also determined the factors affecting the patient safety activities of nurses. This cross-sectional study included 189 nurses from four hospitals in South Korea. The survey was conducted from October to December 2019 using self-report questionnaires including items on socio-demographic characteristics, just culture, empowerment, and patient safety activities. Data were analyzed using descriptive statistics, *t*-test, ANOVA, Pearson’s correlation coefficient, and multiple linear regression analysis with SPSS/WIN 25.0. Patient safety activities correlated positively with just culture (r = 0.369, *p* < 0.000) and empowerment (r = 0.38, *p* < 0.000). Additionally, empowerment (β = 0.213, *p* = 0.001) and just culture (β = 0.442, *p* < 0.000) had statistically significant effects on patient safety activities and explained about 19.5% of the variance in patient safety activities (F = 16.170, *p* < 0.001). The patient safety at medical institutions can be improved by cultivating a work environment that embraces a just culture and empowers nurses.

## 1. Introduction

Despite efforts over the past few decades, patient safety remains an important topic of interest because of the rate of safety errors in the medical environment [1]. During this period, the importance for medical institutions to develop a culture centered on support and learning, in order to improve patient safety, was established [2]. The present focus should be on a more fundamental part of building patient safety culture, related to the psychological aspects of its members. In this regard, Edmonson [3] explained how the psychological safety of members in an organization induces organizational learning, innovation, and growth. It is necessary to pay attention to psychological safety in relation to patient safety in the hospital environment. Moreover, it is crucial to pay attention to research that indicates achievement of innovation and development based on the psychological safety of members in any organization [4].

Regarding the psychological safety of members, it is essential to understand the notion of “just culture” related to error reporting in an organization. In the early stage of studying patient safety culture, the concept of just culture appeared as a sub-concept [5]. In various disciplines that recognize the importance of safety, including aviation, mining, and healthcare, a just culture now denotes the idea of creating a responsible culture by shifting its focus from errors and results to system design and behavior choices [6].

The World Health Organization (WHO) proposed a strategy to define the clear boundary and distinction between medical errors and medical negligence to establish a just culture [7]. Furthermore, the WHO defines just culture as a concept that recognizes the complexity of situations and events and acknowledges that most patient safety failures occur due to weak systems.

According to an integrated review reported in 2021, healthcare organizations must recognize the importance of a just culture in promoting patient safety, develop interventions to improve the just culture, increase error and near-miss reporting, and enhance learning opportunities [5]. Therefore, the present study determined whether nurses’ awareness of the level of the just culture influences their patient safety activities.

Unlike organizational factors such as the just culture, several studies have examined the relationship between personal factors of nurses such as empowerment and patient safety activities [8]. In a randomized controlled trial with 60 nurses, nurses attending an empowerment program showed high indicators related to patient safety performance [9].

Another study of intensive care unit nurses showed that their structural empowerment was significantly statistically correlated with their perception of patient safety environments, thereby supporting the professional empowerment of nurses [10]. Additionally, a study of 262 operating room nurses reported that the higher the level of empowerment of operating room nurses, the greater the impact of patient safety culture awareness on safety management activities [11]. The empowerment of nurses is essential for patient safety because it encourages them to assert themselves through empowerment, and behaviors related to improving patient safety increase when nurses are encouraged to raise their voices in situations that threaten patient safety [9]. Ample studies have explored the concept of empowerment in the context of patient safety in the previous decade. Therefore, empowerment is the power of nurses to influence the behavior of people around them in relation to patient safety [12].

Few studies have considered just culture and empowerment jointly in relation to patient safety activities of nurses. In other words, a study confirming the relationship between the just culture of the organization to which the nurses belong, and the patient safety activities based on their empowerment will be relevant in establishing a patient safety culture and improving the quality of nursing services. Therefore, this study determined the effects of nurses’ perceptions of a just culture and empowerment on their patient safety management.

## 2. Methods

### 2.1. Study Setting

This study aimed to (a) examine the hospital nurses’ perceptions of a just culture, empowerment, and patient safety activities, (b) explore the correlations among hospital nurses’ perceptions of a just culture, empowerment, and patient safety activities, and (c) identify the factors affecting the patient safety activities of nurses.

**Figure 1:**
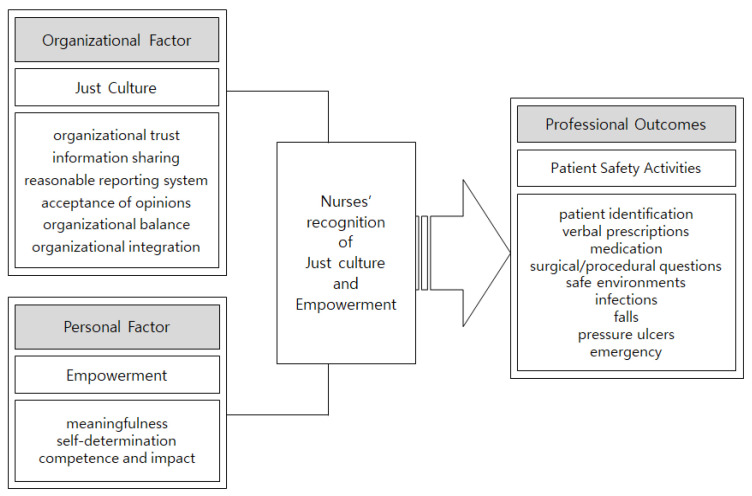
Conceptual framework of this study.

This study used a cross-sectional research design to examine the effects of hospital nurses’ perceptions of a just culture and empowerment on patient safety activities. Figure 1 shows the conceptual framework of this study.

### 2.2. Data Collection

This study was conducted on nurses with more than one year of clinical experience employed at four general hospitals in Seoul and the metropolitan area. The purpose of the study and voluntary nature of participation were explained to the nurses. Informed consent was obtained from those who volunteered to participate in the study. Data were collected from willing nurses between October and December 2019. The Institutional Review Board approved the overall study protocol (IRB No. 2019-08-008-005).

The required sample size for this study was estimated to be 172 participants using G*Power 3.1.9.2 (a free open source program, developed at Heinrich Heine University Düsseldorf, Düsseldorf, Germany) for a linear multiple regression model (α = 0.05, 1 − β = 0.95, effect size f^2^ = 0.15, number of predictors = 10). About 200 individuals were surveyed; after accounting for the dropout rate, data from 189 nurses were finally analyzed.

### 2.3. Measures

#### 2.3.1. Just Culture

The Korean Just Culture Assessment Tool (K-JCAT), a 24-item questionnaire, was used to measure the perception of just culture in this study. The Just Culture Scale [13] was translated into Korean by Lee [14]. The K-JCAT contains six factors: “organizational trust,” “information sharing,” “reasonable reporting system,” “acceptance of opinions,” “organizational balance,” and “organizational integration.” It is rated on a 5-point Likert scale; the higher the score, the higher the just cultural characteristics of the hospital as recognized by the nurse. Cronbach’s alpha was 0.88 in Lee’s study [14], and 0.72 in this study.

#### 2.3.2. Empowerment

Empowerment was measured using the Psychological Empowerment Scale developed by Spritzer [15]. It is rated on a 5-point Likert scale and comprises 16 items and 4 sub-variants (meaningfulness, self-determination, competence, and impact). The higher the score, the higher the empowerment level. Examples of items from the empowerment measurement tool include: “I think age work is an important task in our department” and “I think I’m recognized as an able person in the workplace.” Cronbach’s alpha was 0.86 in Choo’s study [16] and 0.93 in the present study.

#### 2.3.3. Patient Safety Activities

The instrument to assess patient safety activities was developed by Lee [17] and modified and supplemented by Lee [18]. The tool consists of 40 items on the following patient safety activities of the nurse: patient identification (7 items), verbal prescriptions (3 items), medication (7 items), surgical/procedural questions (4 items), safe environments (3 items), infections (3 items), falls (3 items), pressure ulcers (3 items), and emergencies (7 items). The items are rated on a 5-point Likert scale. Higher scores indicate a more positive perception of the nurse’s patient safety activities. Cronbach’s alpha was 0.95 in Lee’s study [17] and 0.92 in this study.

### 2.4. Data Analysis

Data analysis was performed using SPSS 25.0 (SPSS Inc., Somers, NY, USA). Participants’ general characteristics were analyzed using descriptive statistics including means and standard deviations. Just culture, empowerment, and patient safety activities were analyzed using *t*-test and ANOVA according to the participants’ general characteristics. Scheffe’s method was used for post-hoc analysis. Pearson’s correlation coefficient was used to examine the correlations between the participants’ perception of a just culture, empowerment, and patient safety activities. Multiple linear regression analysis was performed to determine the factors affecting patient safety management activities.

## 3. Results

### 3.1. Participants’ General Characteristics

Concerning the general characteristics of the participants, 179 (94.71%) participants were female and 10 (5.29%) were male. About 113 (59.79%), 60 (31.75%), 10 (5.29%), and 6 (3.17%) participants were aged between 20 to 29 years, 30 to 39 years, 40 to 49 years, and 50 or above, respectively. Concerning academic background, 135 (71.43%) participants had a bachelor’s degree, 33 (17.46%) had a master’s degree, 20 (10.58%) were nursing specialists, and one was a doctor (0.53%). About 147 participants were unmarried (77.78%) and 42 were married (22.22%). The most common position was staff nurse (107; 56.61%), followed by charge nurse (72; 38.10%) and head nurse (10; 5.29%). Concerning the department, 79 (41.80%) nurses were posted in the ward, 49 (25.93%) nurses in the intensive care unit, 45 (23.81%) nurses in the emergency center, and 6 (3.17%) were in the outpatient department. About 66 (34.92%) nurses had a total clinical experience of between one and three years, 51 (26.98%) had between three and six years, 31 (16.40%) had between six and 10 years, and 41 (21.69%) had a clinical experience of more than 10 years. Concerning their current department experience, 69 (36.51%) nurses had experience of one to three years, 55 (29.10%) had between three to six years, 30 (15.87%) had experience of six or more years, 21 (11.11%) had experience of less than one year, and 14 (7.41%) had more than 10 years’ experience.

About 83 (43.92%), 60 (31.75%), 26 (13.76%), and 20 (10.58%) nurses reported working overtime one day every week, two to three days every week, three to four days every week, and every day, respectively.

### 3.2. Just Culture, Empowerment, and Patient Safety Activities of Participants

The mean score of nurses’ perceptions of a just culture was 2.95 ± 0.25 out of 5. The mean score on empowerment was 3.59 ± 0.51 out of 5, while the mean score on patient safety activities was 4.22 ± 0.43 out of 5 (Table 1). Table 2 presents the differences in just culture, empowerment and patient safety activities according to their general characteristics. Concerning just culture, statistically significant differences were found in age (F = 11.547, *p* < 0.001), education (F = 5.347, *p* < 0.001), position (F = 12.390, *p* < 0.001), department (F = 6.753, *p* < 0.001), clinical experience (F = 9.911 *p* < 0.001), and current department experience (F = 4.413, *p* = 0.005). Empowerment showed a statistically significant difference according to position (F = 4.818, *p* = 0.009); empowerment of head nurses was found to be higher than that of staff and charge nurses. Patient safety activities showed a statistically significant difference according to gender (F = 3.13, *p* = 0.044); male nurses had higher patient safety activities than female nurses.

### 3.3. Correlations among Nurses’ Perceived Just Culture, Empowerment, and Patient Safety Activities

Just culture showed significant positive correlations with empowerment (r = 0.427, *p* < 0.001) and patient safety activities (r = 0.369, *p* < 0.001). Additionally, empowerment showed a significant positive correlation with patient safety activities (r = 0.380, *p* < 0.001) (Table 3).

### 3.4. Factors Influencing Patient Safety Activities

To examine the effects of various variables on patient safety activities, assumptions of the regression analysis on independent variables were verified. Just culture, empowerment, and patient safety activities of the participants were placed in the regression model. Since the Durbin–Watson statistic was 1.916 in the autocorrelation verification of the error, there was no problem of autocorrelation. In the multicollinearity check, the tolerance limit was 0.1 or more and the variance expansion factor (VIF) was not more than 10, confirming the independence of the predictive variables. Additionally, the assumptions of linearity, normality, and homoscedasticity to meet the residual assumptions were satisfied. Empowerment (β = 0.213, *p* = 0.001) and just culture (β = 0.442, *p* < 0.000) were subsequently identified as variables that had statistically significant effects on patient safety activities, and explained about 19.5% of the variance in patient safety activities (Table 4).

## 4. Discussion

By adopting a just culture, an organization can shift its focus from judging errors and consequences to root causes. It allows more productive discussions about system design and action choices beyond the interest in who made an error [6].

Therefore, this study examined the degree of just culture, empowerment, and patient safety activities of hospital nurses and determined the effects of a just culture and empowerment on patient safety activities to improve the level of patient safety activities.

The mean score of nurses’ perceptions of a just culture was 2.95 out of 5. The just culture score in this study was lower than 3.5, which was reported in a 2021 study on nurses using the JCAT tool [19]. The reason for this difference is that Lee’s study reflects the characteristics of one of the largest hospitals in Korea; the size of a hospital is closely related to the nursing work environment. Nursing organizations differ depending upon hospital size [20], and nurses’ perceptions of organizational policy differ based on the metropolitan area and hospital size [21]. Since little research has been conducted with nurses, it is difficult to compare the findings with previous studies. However, the results correspond with those reported by a study on Irish Air Force pilots [22]; the scores reported are lower than the scores of US nursing students [23]. The results of the present study suggest that nurses in general hospitals need to make efforts to improve their awareness about the characteristics of a just culture.

The average scores on empowerment and patient safety activities were 3.59 and 4.22 out of 5, respectively. Empowerment scores in the present study were similar to those of the previous studies on nurses employed in six hospitals (3.48) [24] and nurses in the operating room (3.67) [11]. The results on patient safety activities were similar to those of general hospital operating room nurses (4.22) [11] but higher than those of nurses in hospitals of various sizes (3.75) [21].

The three variables of just culture, empowerment, and patient safety activities showed significant positive intercorrelations. The results showed that the higher the nurses’ perceived level of just culture and level of empowerment, the better the performance on patient safety activities. Since research in this regard is insufficient, the discussion on the comparison of results with previous studies is limited. However, it is evident that if a hospital lacks a just culture environment and does not empower nurses, the patient safety activities of the nurses may be low. Therefore, it is necessary to build a just culture that can strengthen patient safety activities. Additionally, the empowerment of nurses affects their attitudes and behaviors, enabling superior decision-making in the clinical field and improving the overall quality of nursing care provided to patients. Therefore, medical institutions must endeavor to empower nurses. According to the present results, there was a difference in the level of just culture and empowerment according to the position of nurses. Therefore, if the staff nurse, not the manager, is highly aware of the just culture and plans to be empowered, it will help the nurses improve their patient safety activities.

The results of this study that there is a difference in the perception of just culture according to the nurses’ clinical experience are consistent with the results of Lee’s study [19]; however, this study also found significant differences by age, education, position, and department. The difference in empowerment according to general characteristics was statistically significant according to position, which was consistent with the results of a previous study [24]. Patient safety activities showed differences according to gender and found that male nurses had higher patient safety activities than female nurses. However, in this study, the number of male nurses was small. Therefore, future studies must verify this through repeated studies.

In this study, multiple regression analysis showed that recognition of a just culture and empowerment were the factors affecting patient safety activities, with a total explanatory power of 19.6%. It is difficult to discuss the degree of influence of these two factors in this study because no previous studies on patient safety activities including these variables have been conducted. However, to perform patient safety activities efficiently, it is necessary to establish a just culture in medical institutions and continuously increase the empowerment of nurses. Furthermore, it is necessary to improve the organizational climate related to patient safety, such as the organizational culture related to safety reporting systems, and to improve the system sequentially by checking the system step by step. The transition to a just culture is a slow process that takes years, with hospital policy expansion to integrate cultural principles being the first step [25]. In the healthcare system, patient care results include both organizational system design and individual behavior choices [26]. Thus, a just culture does not mean a blame-free culture, but rather a culture of balanced accountability [25].

What we should not overlook, however, is that the form of the just culture should change in terms of system access and not in terms of retribution on individuals. Dekker and Breakey [27] emphasized the following characteristics of restorative justice: Unlike retributive justice, which focuses on individual errors or violations, restorative justice requires a healing process in response to the harm caused by errors or violations. Moreover, approaches to justice and responsibility are more comprehensive than in retributive approaches.

In particular, an important difference in restorative justice is characterized by listening to everyone affected by errors or problems. However, in retributive justice, only employees related to problems or errors are targeted [27].

Restorative justice allows one to listen to multiple accounts and look at what to do to restore damaged trust and relationships. Therefore, it aims to promote dialogue between actors and surrounding communities (e.g., colleagues) and prevent the collapse of relationships and trust that may arise through sanctions and punishment [28].

More strictly speaking, a just culture should be based on the achievement of substantive, procedural, and restorative justice—thereby improving safety. On the basis of this restorative justice culture, it will be possible for hospital members, including the nurses we described earlier, to work in a state of psychological safety.

In this study, unit managers were considered to have a high awareness of just culture and empowerment because of high confidence in their work and high awareness of responsibility toward patients based on various experiences of nursing practice. This study determined the effects of hospital organizations’ just culture and empowerment on patient safety activities of nurses, and it is relevant in that it confirmed that a just culture and empowered nurses have significant effects on patient safety activities. This study has certain limitations. First, because data was collected using self-report questionnaires from nurses with more than twelve months of experience from only four hospitals, the results have limited generalizability. Second, as this was a cross-sectional study, the causal relationship between the variables could not be inferred.

## 5. Conclusions

This study aimed to provide a basis for establishing a safe organizational culture in hospitals by identifying the relationship between hospital nurses’ perception of a just culture, empowerment, and patient safety activities. We further determined the effect of these two variables on patient safety activities. The results of the present study showed that just culture and empowerment are factors that affect patient safety activities. An organizational culture that has the characteristics of a just culture should be built to improve patient safety activities of hospital nurses. Additionally, management efforts are needed to increase the empowerment of nurses when they engage in professional nursing practices along with various intervention programs and policies for cultural improvement.

## Figures and Tables

**Table 1:** Level of Just culture, Empowerment, and Patient Safety Activities.

Variables	Mean ± SD	Range	
		Min.	Max.
Just Culture	2.95 ± 0.25	1	5
Empowerment	3.59 ± 0.51	1	5
Patient Safety Activities	4.22 ± 0.43	1	5

**Table 2:** Just culture, Empowerment and Patient Safety Activities by General Characteristics (*n* = 189).

Characteristics	Category	*n*	Just Culture		Empowerment		Patient Safety Activities	
			M ± SD	t or F (*p*) Scheffé	M ± SD	t or F (*p*) Scheffé	M ± SD	t or F (*p*) Scheffé
Gender	Male	10	2.99 ± 0.28	0.40 (0.456)	3.91 ± 0.70	1.98 (0.083)	4.5 ± 0.08	3.13 (0.044)
	Female	179	2.95 ± 0.24		3.58 ± 0.49		4.20 ± 0.03	
Age (years)	20–29 ^a^	113	2.88 ± 0.23	11.547 (<0.001) c > a	3.57 ± 0.50	3.398 (0.091)	4.23 ± 0.42	0.897 (0.444) -
	30–39 ^b^	60	3.04 ± 0.23		3.55 ± 0.51		4.16 ± 0.42	
	40–49 ^c^	10	3.20 ± 0.18		3.89 ± 0.42		4.39 ± 0.48	
	≥40 ^d^	6	3.13 ± 0.20		4.10 ± 0.57		4.23 ± 0.57	
Education	Diploma ^a^	20	2.89 ± 0.26	5.347 (0.001)	3.69 ± 0.41	0.899 (0.443)	4.07 ± 0.44	0.952 (0.417)
	Bachelor ^b^	135	2.93 ± 0.24		3.56 ± 0.54		4.23 ± 0.43	
	Master ^c^	33	3.10 ± 0.23		3.69 ± 0.43		4.27 ± 0.43	
	PhD ^d^	1	3.27		3.75		4.12	
Marital status	Single	147	2.90 ± 0.23	−6.597 (0.403)	3.53 ± 0.47	−3.242 (0.149)	4.20 ± 0.42	−0.810 (0.152)
	Married	42	3.16 ± 0.21		3.82 ± 0.57		4.26 ± 0.47	
Position	Staff nurse ^a^	107	2.91 ± 0.22	12.390 (<0.001) c > a, b	3.57 ± 0.54	4.818 (0.009) c > a, b	4.23 ± 0.42	0.552 (0.577) -
	Charge nurse ^b^	72	2.98 ± 0.26		3.56 ± 0.55		4.18 ± 0.43	
	Unit manager ^c^	10	3.28 ± 0.16		4.08 ± 0.47		4.33 ± 0.54	
Department	Ward ^a^	79	2.88 ± 0.25	6.753 (<0.001) e > a, c	3.58 ± 0.55	1.082 (0.367)	4.19 ± 0.41	1.692 (0.154)
	ICU ^b^	49	2.91 ± 0.20		3.54 ± 0.39		4.14 ± 0.46	
	ER ^c^	45	3.06 ± 0.22		3.68 ± 0.56		4.35 ± 0.39	
	OPD ^d^	6	3.21 ± 0.28		3.35 ± 0.37		4.16 ± 0.55	
	others	10	3.07 ± 0.23		3.77 ± 0.53		4.27 ± 0.44	
Clinical experience (years)	1–≤ 3 ^a^	66	2.87 ± 0.20	9.911 (<0.001) d > a, b	3.54 ± 053.	2.210 (0.088)	4.25 ± 0.38	0.621 (0.602)
	3–≤ 6 ^b^	51	2.90 ± 0.21		3.56 ± 0.51		4.16 ± 0.49	
	6–10 ^c^	31	3.02 ± 0.22		3.53 ± 0.50		4.19 ± 0.39	
	≥10 ^d^	41	3.11 ± 0.29		3.78 ± 0.46		4.25 ± 0.44	
Current department experience	1–≤ 3 ^a^	21	2.98 ± 0.29	4.413 (0.005) d > b	3.70 ± 066.	1.075 (0.361)	4.19 ± 0.49	0.606 (0.612)
	3–≤ 6 ^b^	69	2.90 ± 0.22		3.55 ± 0.52		4.26 ± 0.40	
	6–10 ^c^	55	2.93 ± 0.20		3.54 ± 0.45		4.16 ± 0.46	
	≥10 ^d^	44	3.06 ± 0.29		3.68 ± 0.49		4.23 ± 0.40	
Overtime days per week	0–1 ^a^	83	2.99 ± 0.21	2.282 (0.081)	3.61 ± 049	0.172 (0.915)	4.26 ± 0.44	0.759 (0.518)
	2–3 ^b^	60	2.92 ± 0.28		3.56 ± 0.55		4.16 ± 0.42	
	3–4 ^c^	26	2.88 ± 0.23		3.58 ± 0.45		4.23 ± 0.46	
	Everyday ^d^	20	3.01 ± 0.28		3.64 ± 0.58		4.17 ± 0.34	

ICU: Intensive Care Unit; ER: Emergency Room; OPD: Outpatient Department.

**Table 3:** Correlations among Nurses’ Perceived Just Culture, Empowerment, and Patient Safety Activities.

Variables	Just Culture (*p*)	Empowerment (*p*)	Patient Safety Activities (*p*)
Just Culture	1		
Empowerment	0.427 (< 0.000)	1	
Patient Safety Activities	0.369 (< 0.000)	0.380 (< 0.000)	1

**Table 4:** Factors Influencing Patient Safety Activities.

Variables	*B*	*SE*	β	*t*	*p*
(Constant)	2.345	0.367		6.384	0.000
Gender	−0.209	0.127	−0.108	−1.638	0.103
Just Culture	0.442	0.124	0.257	3.547	0.000
Empowerment	0.213	0.061	0.254	3.477	0.001
*F*-value		16.170			
*p*		< 0.001			
*R* ^2^		0.208			
Adj *R*^2^		0.195			

## Data Availability

The data presented in this study are available on request from the corresponding author.
